# Mr. Thackeray, on a Case of Spina Bifida

**Published:** 1800-02

**Authors:** Frederic Thackeray

**Affiliations:** Cambridge


GENTLEMEN,

IF you think the following case of Spina Bifida worthy of notice in your valuable publication, you have the liberty of inserting it.

I am, with great respect,

Your's, very obediently,

FREDERIC THACKERAY.

*Cambridge,*

*Oct 26, 1799.*

---

Mrs. Z. of this place, was delivered of a boy on the 20th of August, perfect, in every respect, except about the loins, where there appeared a vesication, which seemed to have been burst during the child's passage through the pelvis, and from which came a copious serous discharge, tinged with blood. In the course of ten days it assumed more the colour of the skin, discharging very little, and rising into a tumour of the size and shape of a small mushroom, concave on the top.

The child thrived very much, but expressed signs of pain, and tossed about its arms, when the tumour was pressed upon. Its legs it scarce ever moved, till the day on which it died, which was the thirtieth from its birth, when this swelling ulcerated and became flat, discharging a fluid resembling the tears. For some hours he now moved his eyes briskly, was in great agony, his whole body shrunk; and after a succession of convulsive fits, affecting only his face and arms for a few hours, he died.

Dissection.—The spinous processes of the lumbar vertebræ were wanting. The spinal marrow which passes through them and the tumour were removed together; it consisted of a sac, formed where the spinous processes were deficient, communicating upwards by a small orifice with the groove for the spinal marrow, up which I could introduce a probe two inches, and from which came a tea spoonful of purulent matter. From the spinal marrow arose a fungus, making a column in the centre of of the fac, and inferted into the top of the tumour. The fungus thus attached, explained its concave appearance, as the fides had greater liberty to diftend themfelves, and thus, perhaps, the preffure was in fome degree removed from the fpinal marrow, for the fac preffed chiefly on the tranfverfe procefles of the fpine.

This tumour was nourifhed in an unufual manner; there was a fingle veffel coming from the membrane of the fpinal marrow, running unconnected with the body of the fungus, and inferted into the top of it by two branches.

The fungus appeared like thofe we meet with, after the trepan has been ufed, and the dura mater pierced.

Where Nature has been fo deficient, Art can apply no fubftitute. Preffure muft do harm; we might contrive fomething to protect the part from that, and, perhaps, prolong a life that we cannot hope to preferve. In this inftance, the preffure of the diftended fac produced a paralyfis of the lower extremities, which was removed when it was ruptured. I do not think it communicated high up the fpine, as preffure on it only excited crying, and made the infant tofs about its arms. It flept, fucked, and had evacuations as other infants. It was moft quiet when lying on its back, in the hollow formed between the two knees of the nurfe.

The tumour was, probably, perfect in utero, and burft in its paffage; its mother fays, it moved as brifk as any child fhe ever had.

---

